# Does evolution of echolocation calls and morphology in *Molossus* result from convergence or stasis?

**DOI:** 10.1371/journal.pone.0238261

**Published:** 2020-09-24

**Authors:** Livia O. Loureiro, Mark D. Engstrom, Burton K. Lim

**Affiliations:** 1 Hospital for Sick Children SickKids Learning Institute, The Centre for Applied Genomics, Toronto, Ontario, Canada; 2 Department of Natural History, Royal Ontario Museum, Toronto, Ontario, Canada; 3 Department of Ecology and Evolutionary Biology, University of Toronto, Toronto, Ontario, Canada; Museum National d’Histoire Naturelle, FRANCE

## Abstract

Although many processes of diversification have been described to explain variation of morphological traits within clades that have obvious differentiation among taxa, not much is known about these patterns in complexes of cryptic species. *Molossus* is a genus of bats that is mainly Neotropical, occurring from the southeastern United States to southern Argentina, including the Caribbean islands. *Molossus* comprises some groups of species that are morphologically similar but phylogenetically divergent, and other groups of species that are genetically similar but morphologically distinct. This contrast allows investigation of unequal trait diversification and the evolution of morphological and behavioural characters. In this study, we assessed the role of phylogenetic history in a genus of bat with three cryptic species complexes, and evaluated if morphology and behavior are evolving concertedly. The Genotype by Sequence genomic approach was used to build a species-level phylogenetic tree for *Molossus* and to estimate the ancestral states of morphological and echolocation call characters. We measured the correlation of phylogenetic distances to morphological and echolocation distances, and tested the relationship between morphology and behavior when the effect of phylogeny is removed. Morphology evolved via a mosaic of convergence and stasis, whereas call design was influenced exclusively through local adaptation and convergent evolution. Furthermore, the frequency of echolocation calls is negatively correlated with the size of the bat, but other characters do not seem to be evolving in concert. We hypothesize that slight variation in both morphology and behaviour among species of the genus might result from niche specialization, and that traits evolve to avoid competition for resources in similar environments.

## Introduction

Studies of character evolution help illustrate the relative importance of speciation rates, extinction selectivity, as well as ecological and genomic factors in macroevolution [1, 2]. By determining the ancestral states of characters and tracking subsequent change over time, we can examine the morphological and ecological differences among species to better understand speciation processes [3]. The distribution of character states in a group may evolve by several routes. Shared character states might be the result of evolutionary stasis, in which morphology or behaviour accrue negligible or no change in a lineage over long periods of time. In this scenario, the ancestral state is retained in descendent lineages regardless of the genetic distance and phylogenetic divergence among species [4]. Similar character states might also evolve by convergent evolution, wherein these traits evolve independently in unrelated lineages as a result of adaptation to similar environments or ecological niches [5–8]. Functionally correlated traits might also evolve by concerted evolution, whereby the adaptive values of a specific behaviour depend on a morphological state [9, 10].

A number of diversification processes have been described to explain variation of morphological traits within clades with high divergence rates [11–13]. However, not much is known about these patterns in complexes of cryptic species with low morphological disparity. Both evolutionary conservatism and convergence can underestimate phenotypic divergence, and both mechanisms can produce similar evolutionary outcomes [14, 15]. The study of processes underpinning the evolution of crypsis can only be investigated when species boundaries are well defined. However, because of their similarity, cryptic species are difficult to distinguish based on morphology alone. The precise identification of species within these complexes therefore often requires the study of genetic or behavioural data [16–19]. The mastiff bats of the genus *Molossus* include groups of morphologically similar but genetically distant species, and other groups of species that are morphologically divergent but genetically similar [20–23], which until recently have hindered the resolution of systematic relationships among species of the genus. However, a genomics approach has resulted in a robust phylogeny [24] so that *Molossus* is an excellent case study of the evolution of morphological and behavioural characters to investigate unequal trait diversification in a monophyletic group with variable rates of evolution among lineages.

*Molossus* are mainly Neotropical in distribution from the southeastern United States to southern Argentina, including the Caribbean islands [25]. *Molossus* species are aerial insectivores and are non-migratory, although they have numerous wing adaptations that are associated with high dispersal ability and rapid flight [26–29]. A recent study using Next Generation Sequencing (NGS) [24] yielded thousands of single nucleotide polymorphisms (SNPs) and 14 species of *Molossus* were recognized in the SNPs analyses, including three groups of cryptic species previously identified as *M*. *molossus*, *M*. *currentium*, and *M*. *rufus*: (1—*M*. *molossus*—*M*. *milleri*—*M*. *verrilli*; 2—*M*. *currentium*—*M*. *bondae*; 3—*M*. *rufus*—*M*. *nigricans*—*M*. *fluminensis*). Each cryptic complex is not reciprocally monophyletic, but instead includes morphologically similar species based on characters traditionally used to identify taxa in the genus, such as size, hair patterns, and cranial characters [20, 23].

In *Molossus*, several morphologically similar species (e. g. *M*. *bondae*, *M*. *molossus*, and *M*. *coibensis*) occur in sympatry in the mainland Neotropics and can be distinguished based on their echolocation calls [30], although morphological characters are also necessary for identification. Several of these diagnostic morphological characters are also ecologically and behaviorally important. For example, differences in the infraorbital foramen have been connected to thermoregulation through vasodilation [31] and to sensory acuity of the maxilla in mammals [32]; and the sagittal crest is correlated to bite strength, and consequentially feeding habits [33, 34]. Hair patterns have also been associated with defensive and offensive behaviours [35], mate signaling, and camouflage [36]. Dentition is associated with diet, including mechanical aspects of feeding and processing of diverse food textures [37, 38]. The occipital bone is a curved structure in the rear of the skull perforated by the foramen magnum, through which several nerves (including the spinal cord) and ligaments pass. This bone contributes to the protection of the brain, but character states of this structure do not correlate to phylogenetic data [39].

In bats, phylogenetic relationships may impose constraints on potential echolocation call design within families [40] and genera [30–41] and may explain the differences in call structure within some groups. Conversely, echolocation call frequency might correlate with body size [42], frequency partitioning among species [43], prey size [44], and selective pressures such as foraging strategy and habitat structure [45–47]. Although many hypotheses have been proposed to explain diversity in call design, previous studies support the idea that echolocation is evolutionarily flexible and is constantly adapting to maximize prey detection by adjusting to an optimal aural field of view and novel environments [48, 49].

Echolocation call patterns are generally organized into search, approach, and terminal phases [50]. Search parameters are limiting factors for insect detection and can give information on how the bats optimize their echolocation calls to search for prey [51, 52]. Molossid bats have a long, narrowband search call, a common pattern for insectivorous bats that forage in open areas [30, 53]. A narrow bandwidth concentrates the energy of the signal, which helps in the detection of prey at long distances [54, 55]. In *Molossus*, call designs may vary between two to three echolocation pulses depending on species, starting with a lower-frequency pulse, followed by one or two pulses at successively higher frequencies [30, 56, 57]. This increase of frequencies is hypothesized to allow the detection of a larger number of potential prey sizes and maximize successful capture [30]. Among *Molossus*, echolocation call designs may also vary in duration, harmonics, and structure depending on the species [30, 57–59].

Documenting distinct stereotyped echolocation calls for a group of closely related species would allow us to establish the predominant factors (e.g. phylogenetic stasis, adaptation) involved in evolution of call structure. In this study, we examined traits that varied significantly among some species of *Molossus*, to test the hypothesis that any lack of variability in morphological (i.e., external and cranial features) and behavioral (i.e., echolocation calls) character states is the result of evolutionary stasis. According to this hypothesis, we would expect that variation among morphological and/or echolocation call character states is correlated with phylogenetic relationship. Alternatively, if morphology and/or echolocation call parameters are independent of phylogeny, these traits are most likely evolving stochastically or via local adaptation. In addition, we examined whether morphology and echolocation calls evolve in concert, and the potential association between morphological characters and echolocation call characters states. However, if morphology and echolocation call design are uncorrelated, these suites of traits are likely evolving independently.

## Materials and methods

### Phylogenetic analysis

This study conformed to the animal care and use guidelines of the American Society of Mammalogists [60] and was approved by the Animal Use Committee of the Royal Ontario Museum. Loureiro et al. [24] reconstructed a well-resolved phylogenetic tree of *Molossus* at the species level based on 29,448 filtered SNPs which we used in this study of the evolution of morphology and echolocation calls. The *de novo* alignment comprised 189 samples from 14 recognized species of *Molossus* and representatives of two other genera of molossids, *Promops centralis* and *Eumops auripendulus*, used as outgroups following Ammerman et al. [61] and Gregorin and Cirranello [62]. We used the maximum likelihood phylogeny provided by Loureiro et al. [24] as an initial tree, and individuals were assigned to species. We reconstructed a Bayesian tree using the program SNAPP v1.1.10 [63] implemented in BEAST [64]. We generated the XML file required as input by SNAPP using the Ruby script (snapp_prep.rb) [65]. We ran SNAPP for ten million generations using default priors. Convergence of the runs was assessed through estimated Effective Sample Size (ESS) values and trace plots in Tracer [66]. After removing 10% of the samples as burn-in, we constructed a species tree using TreeAnnotator [67].

### Morphological data

We analyzed 660 specimens from the 14 recognized species of *Molossus* and two outgroup species (S1 Appendix) [24, 68, 69]. The specimens examined included 24 *M*. *alvarezi*; 36 *M*. *coibensis*, 330 *M*. *molossus*, 65 *M*. *aztecus*, 7 *M*. *currentium*, 13 *M*. *bondae*, 3 *M*. *fentoni*, 42 *M*. *pretiosus*, 38 *M*. *fluminensis*, 10 *M*. *nigricans*, 57 *M*. *rufus*, 6 *M*. *sinaloae*, 10 *M*. *milleri*, 8 *M*. *verrilli*, 6 *Eumops auripendulus*, and 6 *Promops centralis*. We also analyzed the holotypes of *M*. *coibensis*, *M*. *bondae*, *M*. *sinaloae*, *M*. *verrilli*, and *M*. *pretiosus*, and photographs of the holotype of *M*. *rufus*. In addition, topotypes of *M*. *alvarezi*, *M*. *milleri*, and *M*. *molossus* were included in our study. Only adults (defined as having closed basioccipital and basisphenoid sutures and complete epiphyseal ossification of metacarpal and phalanx joints) were included in the analyses. Characters were coded based on characteristics from males and bins were determined by examining distributional data and finding natural breaks.

Six morphological characters that have been frequently used to identify species in the genus were analyzed [20, 24, 68] (Tables 1 and 2). For the morphological characters we examined: 1) Size: 0- Small (forearm [FA] length < 37 mm); 1- Medium (FA 37 to 43.5 mm); 2- Large (FA 43.5 to 57 mm); 3- Very large (FA > 57 mm); 2) Dorsal basal hair band: 0- > ½ of the hair length; 1 –No hair band or < ¼ of the length; 3) Shape of upper incisors: 0 –Divergent tips; 1- Thin and elongated with parallel tips; 2 –Pincer-like with convergent tips; 4) Shape of occipital: 0 –Triangular, with undeveloped lambdoidal crest; 1- Rectangular, with well developed lambdoidal crest; 5) Infraorbital foramen: 0 –Laterally directed; 1- Frontally directed; 6)—Size of sagittal crest in males: 0- No sagittal crest; 1- Proportionally undeveloped; 2 –Proportionally developed (Table 1). For the echolocation call parameters, we analyzed six parameters: 1) Call duration: time from the beginning to the end of a call. 0: Long—more than 0.25 sec; 1: medium—0.13 sec to 0.25 sec; 2: Short—less than 0.13 sec.; 2) Lowest call frequency: frequency of lowest call. 0: Low—< 26 kHz; 1: High—> 26 kHz; 3) Highest call frequency: frequency of highest call. 0: Low—< 35 kHz; 1: High—> 35 kHz; 4) Peak Frequency: frequency of maximum energy in a call. 0: Low—< 29 kHz; 1: High—> 29 kHz; 5) Dominant harmonic: call harmonic with the highest energy. 0: first. 1: second; 6) End Slope: slope at the end of the call 0: Absence of downward slope. 1: Presence of downward slope (Table 2).

**Table 1:** Matrix of morphological characters.

	Size	Hair band	Incisors	Occipital	Infraorbital	Sagittal crest
*M*. *fentoni*	0	0	1	0	1	1
*M*. *alvarezi*	2	0	1	0	0	1
*M*. *aztecus*	1	1	2	1	1	2
*M*. *fluminensis*	2	1	2	1	1	2
*M*. *pretiosus*	2	1	2	1	0	2
*M*. *currentium*	1	0	1	0	1	2
*M*. *rufus*	2	1	2	1	1	2
*M*. *bondae*	1	1	2	1	1	2
*M*. *nigricans*	2	1	2	1	1	2
*M*. *molossus*	1	0	1	1	0	1
*M*. *coibensis*	0	1	2	0	0	2
*M*. *milleri*	1	0	1	1	0	1
*M*. *verrilli*	1	0	1	1	0	1
*M*. *sinaloae*	2	0	1	0	0	1
*Promops*	2	1	0	0	0	0
*Eumops*	3	1	0	0	0	0

Characters described in Material and Methods section.

**Table 2:** Matrix of echolocation call parameters.

	Call duration	Minimum Frequency	Maximum Frequency	Peak Frequency	Dominant Harmonic	End Slope
*M*. *fentoni* (n = 0)	?	?	?	?	?	?
*M*. *alvarezi* (n = 57)	2	0	0	1	0	0
*M*. *aztecus* (n = 35)	2	1	1	1	0	1
*M*. *fluminensis* (n = 58)	1	0	0	0	0	0
*M*. *pretiosus* (n = 40)	1	0	0	0	1	0
*M*. *bondae* (Jung et al, 2014)	1	1	1	1	0	1
*M*. *rufus* Jung et al., 2014)	1	0	0	0	0	0
*M*. *currentium* (n = 0)	?	?	?	?	?	?
*M*. *nigricans* (n = 210)	1	1	0	0	0	0
*M*. *molossus* (n = 371)	1	1	1	1	0	1
*M*. *coibensis* (n = 81)	2	1	1	1	0	1
*M*. *milleri* (n = 156)	2	1	1	1	0	0
*M*. *verrilli* (n = 181)	2	1	1	1	0	0
*M*. *sinaloae* (n = 31)	2	1	0	1	0	1
*Promops* (n = 49)	2	0	1	0	0	1
*Eumops*	0	1	1	0	0	1

Characters described in Material and Methods section. n represents the number of calls analyzed per species.

### Echolocation data

Recordings of echolocation calls were obtained in Aruba, Brazil, Belize, Bonaire, Cayman Islands, Curacao, Dominican Republic, Guyana, Mexico, Panama, and Nevis. The permit to conduct scientific research in the Cayman Islands was issued by the Department of Environment of the Cayman Islands; in Aruba by the Fundashon Parke Nacional Arikok; in Belize by the Forest Department, Ministry of Natural Resources and the Environment; in Curacao by the Curacoan Government to CARMABI; in Dominican Republic by the Ministerio de Medio Ambiente y Recursos Naturales; in Guyana by the Environmental Protection Agency; in Nevis by Nevis Island Administration, c/o The Executive Director, Nevis Historical and Conservation Society; in Mexico by the Secretaria de Medio Ambiente y Recuros Naturales; and in Montserrat by the Ministry of Agriculture, Trade, Land, Housing and the Environment. In the other countries there was not an active capture of animals and recordings were made passively.

For species identification in Aruba, Bonaire, Cayman Islands, Curacao, Dominican Republic, Mexico, and Nevis, we captured individuals that were identified to species, measured the forearm length, and released them while recording their calls. The person releasing the bats was about 10 m away from the person recording the calls. Releases were conducted in large open areas and the bats were visually followed in flight until the signal of the calls ended, allowing us to record typical search calls [30, 56, 57]. The calls obtained in Belize, Brazil, and Guyana were from free flying bats, but the species of *Molossus* identified in the call files were also previously caught in mist nests in the respective areas where the calls were recorded. Recordings from Panama were obtained from hand released bats and are described in Gager et al. [57]. Free flying calls were recorded during the first 3 hours after sunset from areas where only one species of *Molossu*s occurs (Aruba, Bonaire, Cayman Islands, Curacao, Dominican Republic, Montserrat, and Nevis) and were compared with the files originating from hand released calls. In total, we obtained echolocation calls for 12 of the 14 species of *Molossus*.

Hand released calls were recorded using Wildlife Acoustics EM3+, Avisoft-UltraSoundGate 116H, and Avisoft-RECORDER USHG. Passive calls were obtained with Wildlife Acoustic SM4BAT FS to a maximum file duration of 15 seconds and initially processed with Kaleidoscope Pro 5 software (Wildlife Acoustics, Inc) followed by manual verification of species. We analysed the search calls in Raven [70] using a Hamming window, FFT = 512, and an overlap of 93%. Faint calls (less than 30 dB relative amplitude) were removed from the dataset.

We measured the duration, peak frequency, minimum frequency, maximum frequency, bandwidth, and pulse interval of a maximum of 10 search calls per bat recording. We calculated both duty cycle (call duration/ pulse interval * 100) and repetition rate (100 ms/ pulse interval). We also analyzed qualitative characteristics of the call, including maximum number of call alternations in a pulse sequence observed for a species, direction of the end slope, number of harmonics of each pulse and noted the harmonic with highest energy. Attack sequences were not included in the analysis because they were recorded in less than 50% of the studied species. Only the harmonic with highest energy for each species was considered for analysis.

Each quantitative measure was plotted individually using the mean and the standard deviation of each species. If the measurements could be divided into two or more groups with no overlapping of mean and standard deviation in the graphs, they were coded and transformed to discrete characters (Tables 1 and 2). Measurements that did not demonstrate variation among species, with means and standard deviation overlapping in the plots (bandwidth, duty cycle, repetition rate) were discarded and not used in further analyses [71–73]. The characters were equally weighted and multi-state characters were treated as unordered. The coded characters were included in a data matrix for analysis, where missing data were denoted as “?” (Tables 1 and 2).

### Data analysis

To determine if the ancestral states were retained in the descendant lineages, we estimated the ancestral characters states for morphological and behavioral characters. Maximum likelihood ancestral reconstructions of the evolutionary path of character state transformation were estimated using the phylogenetic tree recovered from the SNPs analysis. Ancestral states of traits were estimated using Mesquite 3.1 [74] based on a one-parameter model. We used the R package phytools [75] to map characters on the phylogenetic tree. The phylogenetic signal measured by correlations between phylogenetic distances and morphological and echolocation distances were evaluated using the R package phylosignal [76]. We also tested the strength of stochastic Brownian Motion for both morphological and echolocation characters using the package phylosignal [76] by computing the indices of Blomberg’s K and K*, Abouheif’s Cmean, Moran’s I, and Pagel’s Lambda. Results of these simulations can be used to compare the performances of the different methods and interpret values of indices obtained with real trait data, for a given phylogeny [76]. Independent contrasts between quantitative parameters of echolocation calls and quantitative morphological characters were analyzed using the R package phytools [75]. This approach assumes that species have a common history represented by their phylogenetic relationship, and therefore are not independent entities. Independent contrasts analysis removes the phylogenetic components in the correlation of two variables by generating phylogenetically independent variables from the original character values [77]. Correlations between independent contrasts of variables were examined using least squares linear regressions in phytools [75]. To test for correlation between echolocation call parameters, we conducted linear regression analyses of frequency measurements (maximum, minimum, and peak frequencies) versus bandwidth and duration in R 3.6.1.

## Results

### Phylogeny

**Fig 1:**
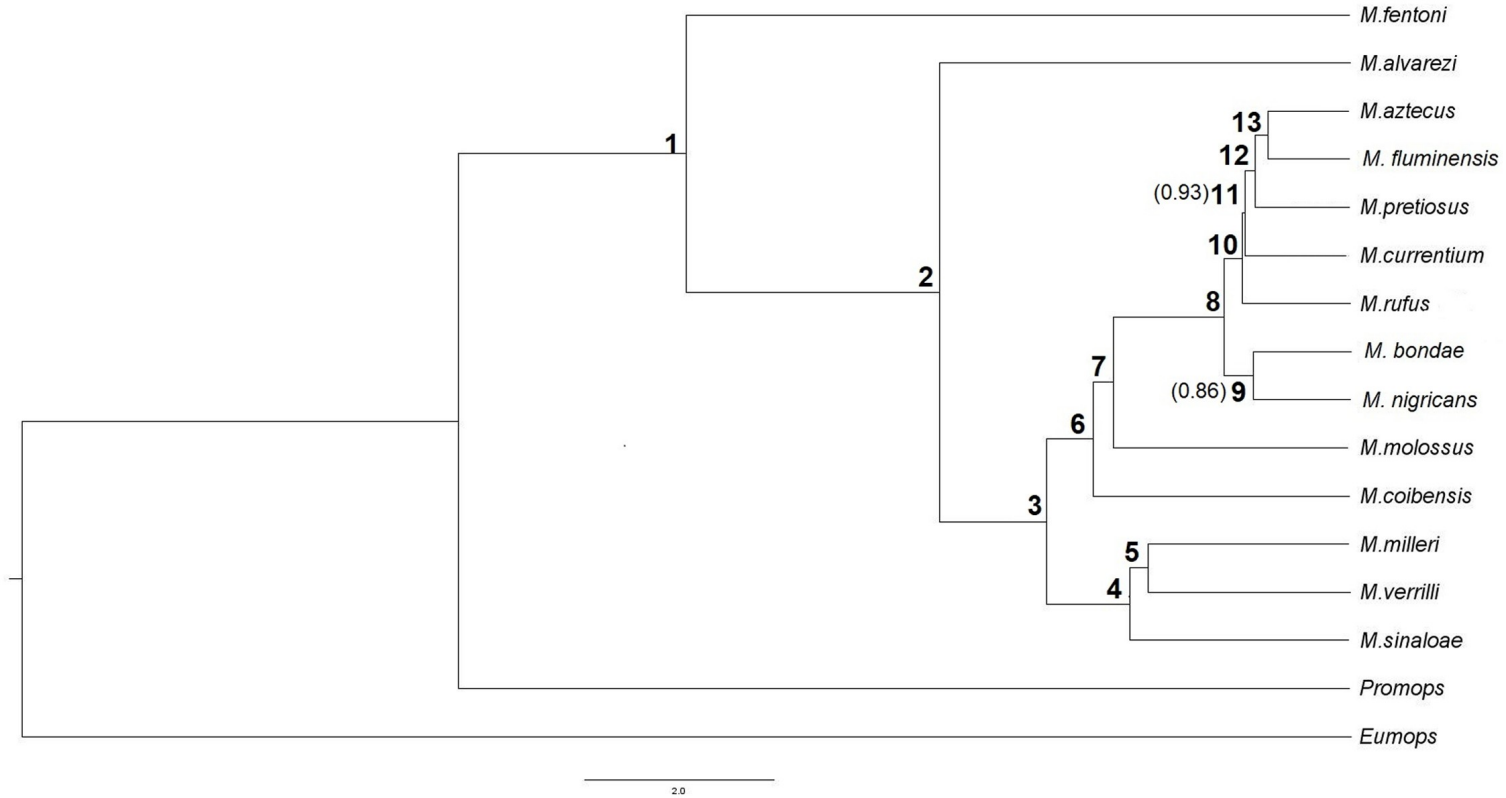
Bayesian species tree of *Molossus* inferred with the DNA sequence alignment of Loureiro et al. (in review). Numbers in parentheses represents the only nodes with posterior probability lower than 95%. The node numbers are described in Table 1.

Bootstrap support for nodes in the Snapp tree among the 14 pre-defined species of *Molossus* is > 85% (Fig 1). The species *M*. *fentoni* from Guyana and Ecuador is the sister group of all other species in the genus. The next species to diverge is *M*. *alvarezi* from the Yucatán Peninsula, Central America and South America, which is the sister group of the remaining species. *M*. *sinaloae* from Western Mexico grouped with the two species from the Greater Antilles, *M*. *milleri* and *M*. *verrilli*. *M*. *coibensis* from Central America and South America appears as the sister species to the clade formed by *M*. *molossus* from South America, Central America, North America and the Lesser Antilles, and the remaining species of the genus (*M*. *bondae*, *M*. *nigricans*, *M*. *aztecus*, *M*. *fluminensis*, *M*. *pretiosus*, *M*. *currentium*, and *M*. *rufus*). In this last group, *M*. *bondae* from Ecuador is the sister species to *M*. *nigricans* from Central America and North America. This clade appears as the sister group of *M*. *aztecus* from Mexico and Brazil, *M*. *fluminensis* from Southeast Brazil and Argentina, *M*. *pretiosus* from Brazil and Nicaragua, *M*. *currentium* from Paraguay, and *M*. *rufus* from Central America, North America and South America.

### Morphological data

The variation of the six morphological characters were consistent with interspecific variation within and among species (Table 1). Some species of *Molossus* have dorsal hair without a small pale band at the base, such as *M*. *rufus* and *M*. *aztecus*. Other species, such as *M*. *molossus* and *M*. *sinaloae*, have dichromatic dorsal hair, with a long pale base comprising more than one-half of the hair length. The upper incisors vary from long and thin (e.g., *M*. *alvarezi*) to short and spatulate (e.g., *M*. *coibensis*). The sagittal crest in males varies from well developed in *M*. *rufus* to proportionally indistinct in *M*. *molossus*. The occipital region can be robust with well developed lambdoidal crests, as in *M*. *aztecus*, to delicate and triangular as in *M*. *fentoni*. The infraorbital foramen might be laterally directed (e.g., *M*. *currentium*) or frontally directed (e.g., *M*. *verrilli*). Many species of *Molossu*s can also be separated by size, varying from small (e.g., *M*. *fentoni*) to medium (e.g., *M*. *bondae*) to large (e.g., *M*. *rufus*).

### Echolocation data

**Fig 2:**
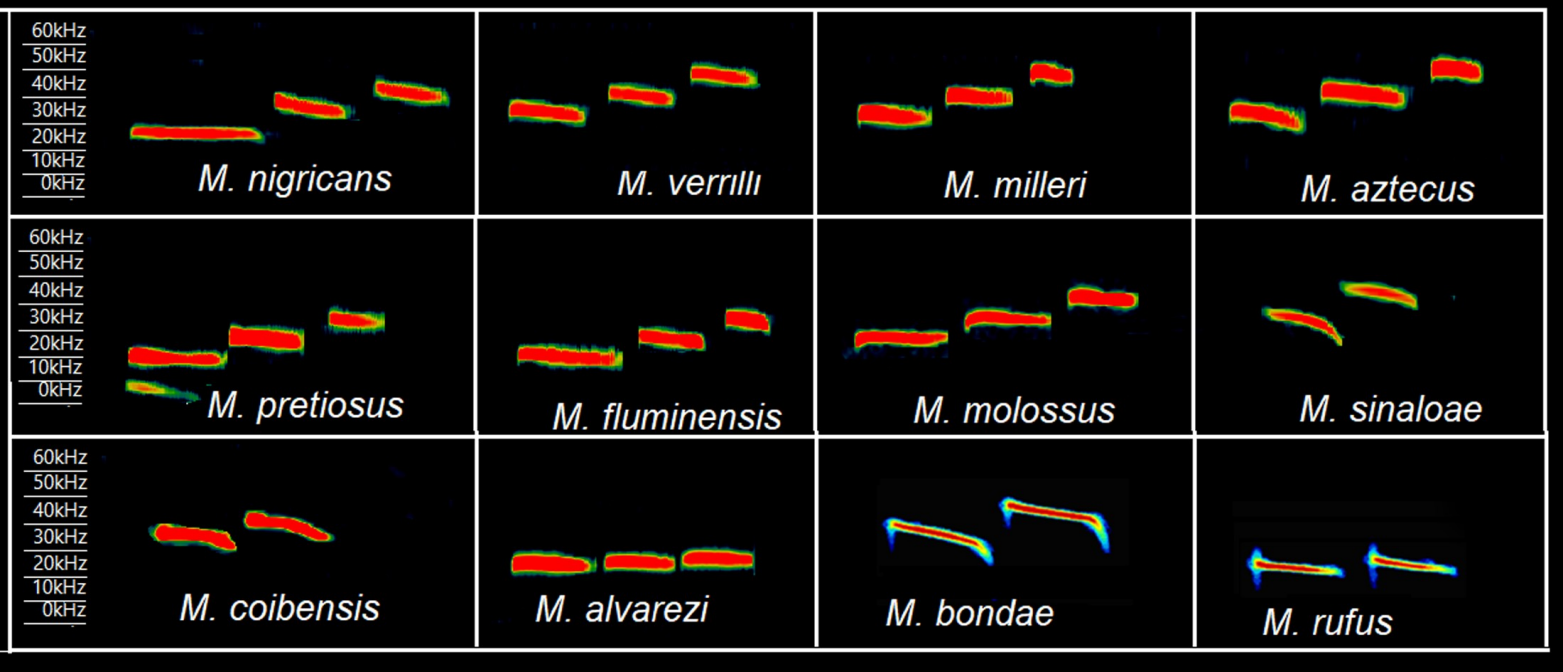
Spectrograms of typical echolocation calls emitted during search phase flight by 12 species of *Molossus*. Calls from M. *bondae* and *M*. *rufus* were taken from Jung et al. (2014).

We recorded a total of 1193 calls from 8 species of *Molossus* and from *Promops centralis* (Fig 2; Table 2). In addition, 81 calls from *M*. *coibensis*, which were published in Gager et al. [57], 31 calls of *M*. *sinaloae* provided from SONOZOTZ project and CONABIO, Mexico were also analyzed (S2 Appendix). Published information on echolocation calls from several species of *Molossus* was also used [30, 65, 78], including information on two species of *Molossus (M*. *bondae and M*. *rufus*) and an outgroup (*E*. *auripendulus*) for which we did not have recordings. Echolocation calls from *M*. *currentium* and the recently described species *M*. *fentoni* [21] were not recorded, and information on the calls of these species is not available in the literature. Therefore, all echolocation characters of *M*. *currentium* and *M*. *fentoni* were coded as unknown.

Calls from the same species that were released by hand and those recorded in the field did not show significant differences in mean values (p <0.05), although free-flying calls increased the standard deviation of the measurements when added to hand-released calls. Free-flying calls appear to be more variable than hand-released calls, but the datasets are not significantly different and were combined for analysis. Only six of 11 parameters of echolocation calls were consistent among species and among different populations within each species. These six parameters were considered for further analyses, which included duration of the call, lowest frequency, highest frequency, peak frequency, highest-energy harmonic, and shape of the end slope.

### Data analysis

**Fig 3:**
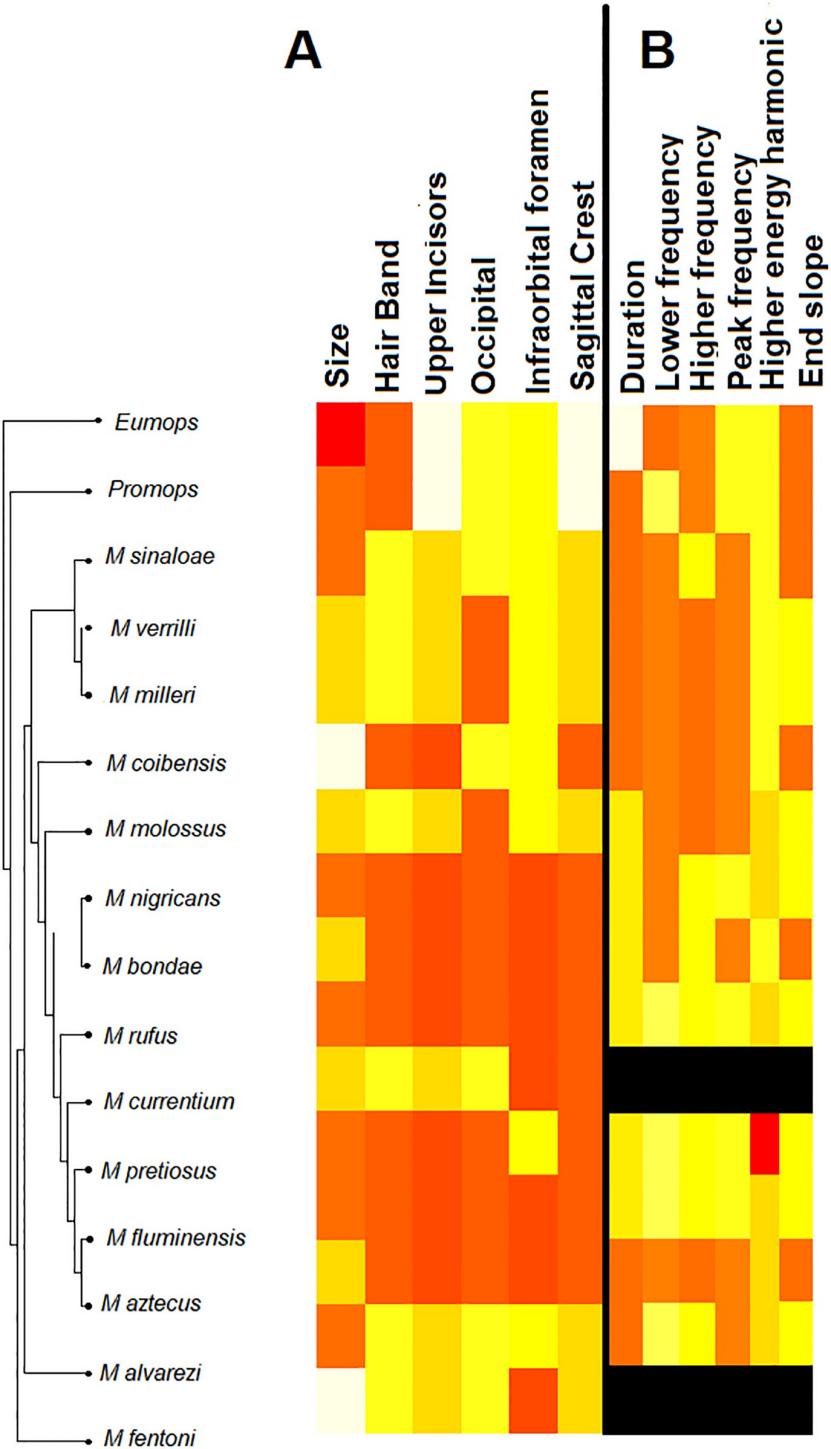
Character mapping on the species tree of *Molossus*. A- Morphological characters; B- Echolocation call characters. Each row represents a character and each color represents a character state. Cells in black represent missing data.

**Fig 4:**
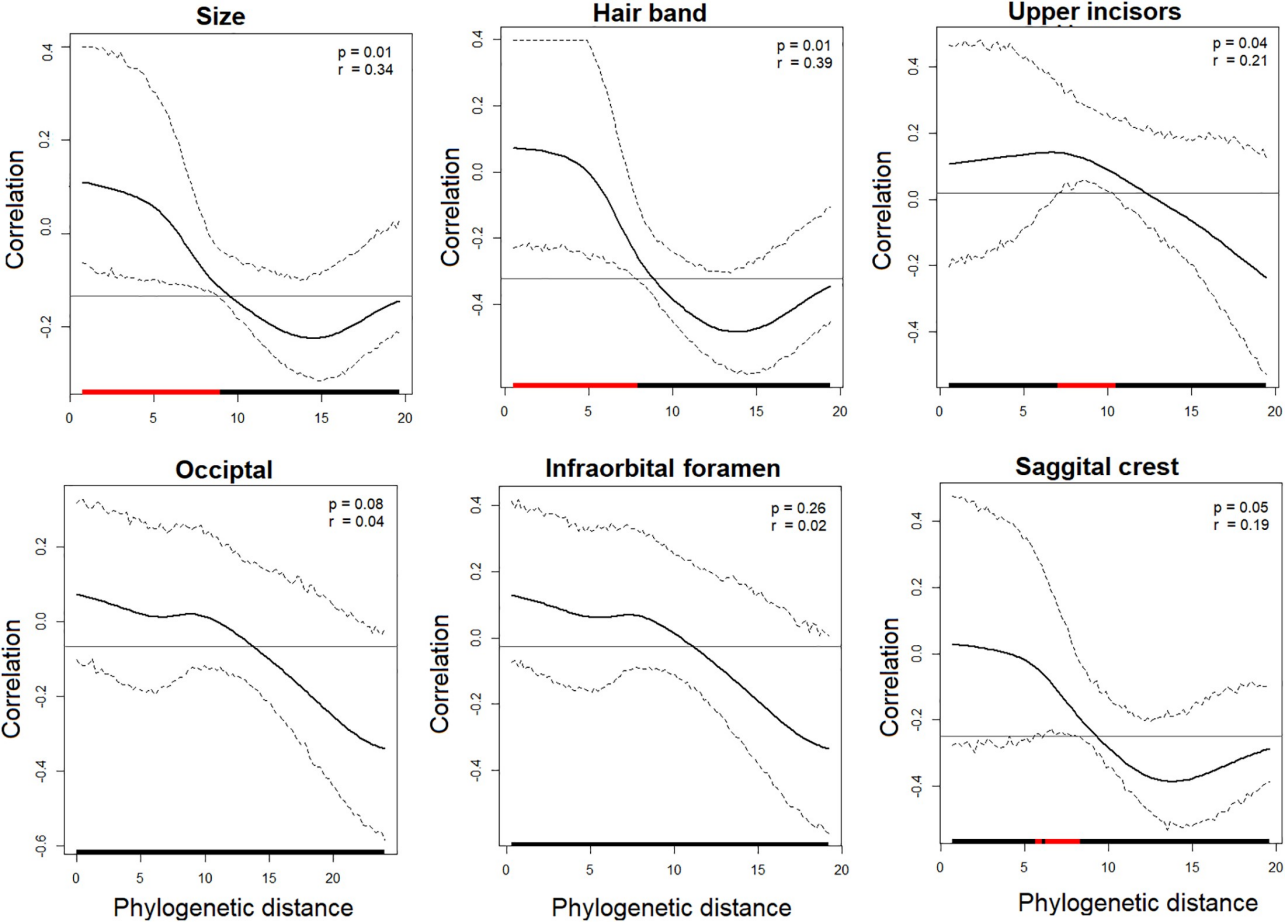
Phylogenetic correlograms between phylogenetic and morphological distances for each morphological character in *Molossus*. The solid bold black line represents the Moran’s I index of autocorrelation, and the dashed black lines represent the lower and upper bounds of the 95% confidence interval. The horizontal black line indicates the expected value of Moran’s I under the null hypothesis of no phylogenetic autocorrelation. The horizontal bar on the x-axis shows whether the autocorrelation is significant (based on the confidence interval): red for significant positive autocorrelation, black for nonsignificant autocorrelation.

**Fig 5:**
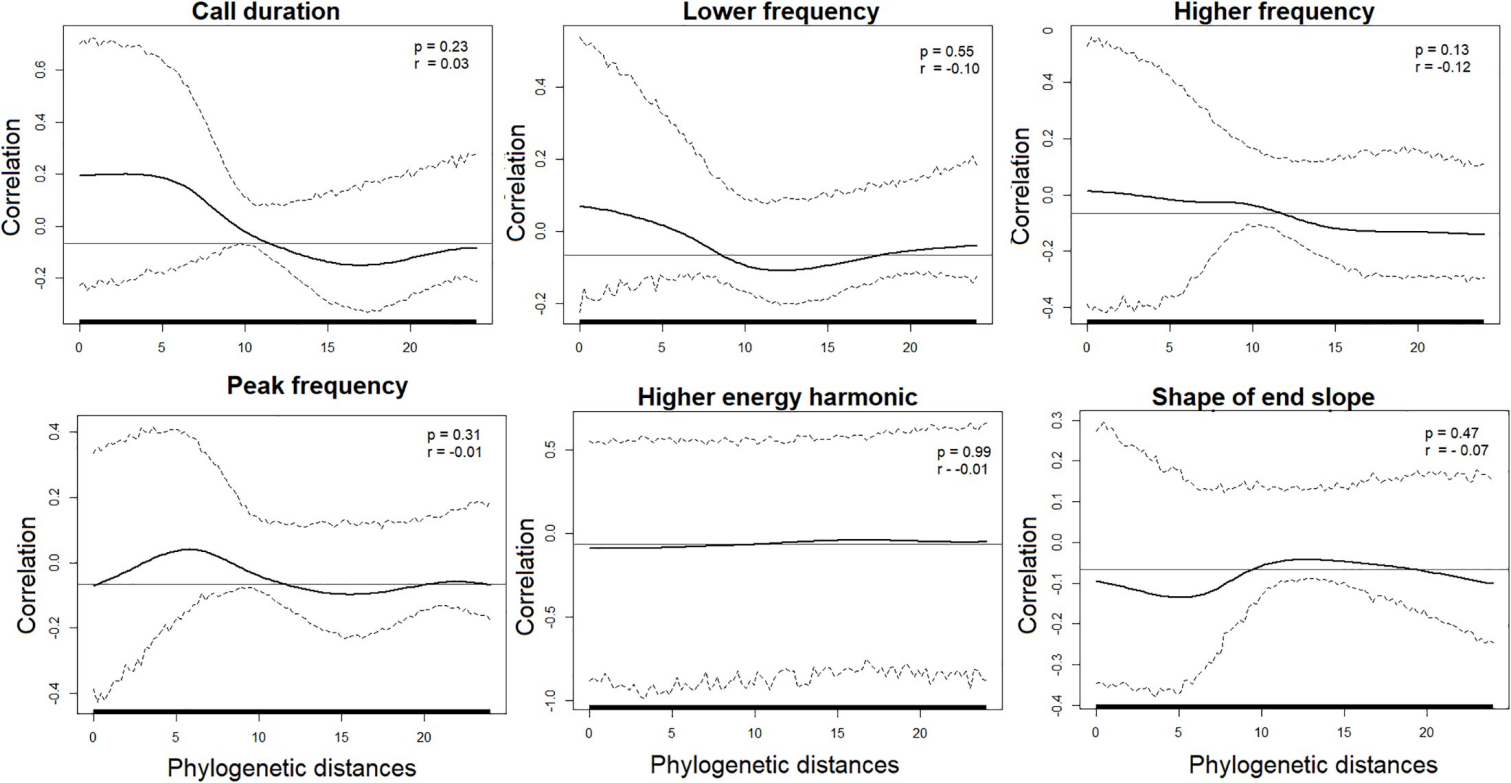
Phylogenetic correlograms between phylogenetic and echolocation call distances for each call parameter in *Molossus*. The solid bold black line represents the Moran’s I index of autocorrelation, and the dashed black lines represent the lower and upper bounds of the 95% confidence interval. The horizontal black line indicates the expected value of Moran’s I under the null hypothesis of no phylogenetic autocorrelation. The horizontal bar on the x-axis shows whether the autocorrelation is significant (based on the confidence interval): red for significant positive autocorrelation, black for nonsignificant autocorrelation.

The six morphological and six echolocation call characters were mapped onto the phylogeny generated by the SNP data (Fig 3). The correlograms showed that size, hair band, and upper incisor shape were positively correlated with phylogenetic distances at p<0.05 (Fig 4). The r values of each of these three correlations varied from 0.21 for the upper incisors to 0.39 for the hair band. The distances of the three remaining morphological characters (occipital shape, infraorbital foramen, and sagittal crest) were not significantly correlated with phylogenetic distances (p>0.05) (Fig 4). The correlogram analysis yielded no correlation between any individual echolocation call parameter and phylogenetic distance (P>0.05) (Fig 5).

**Fig 6:**
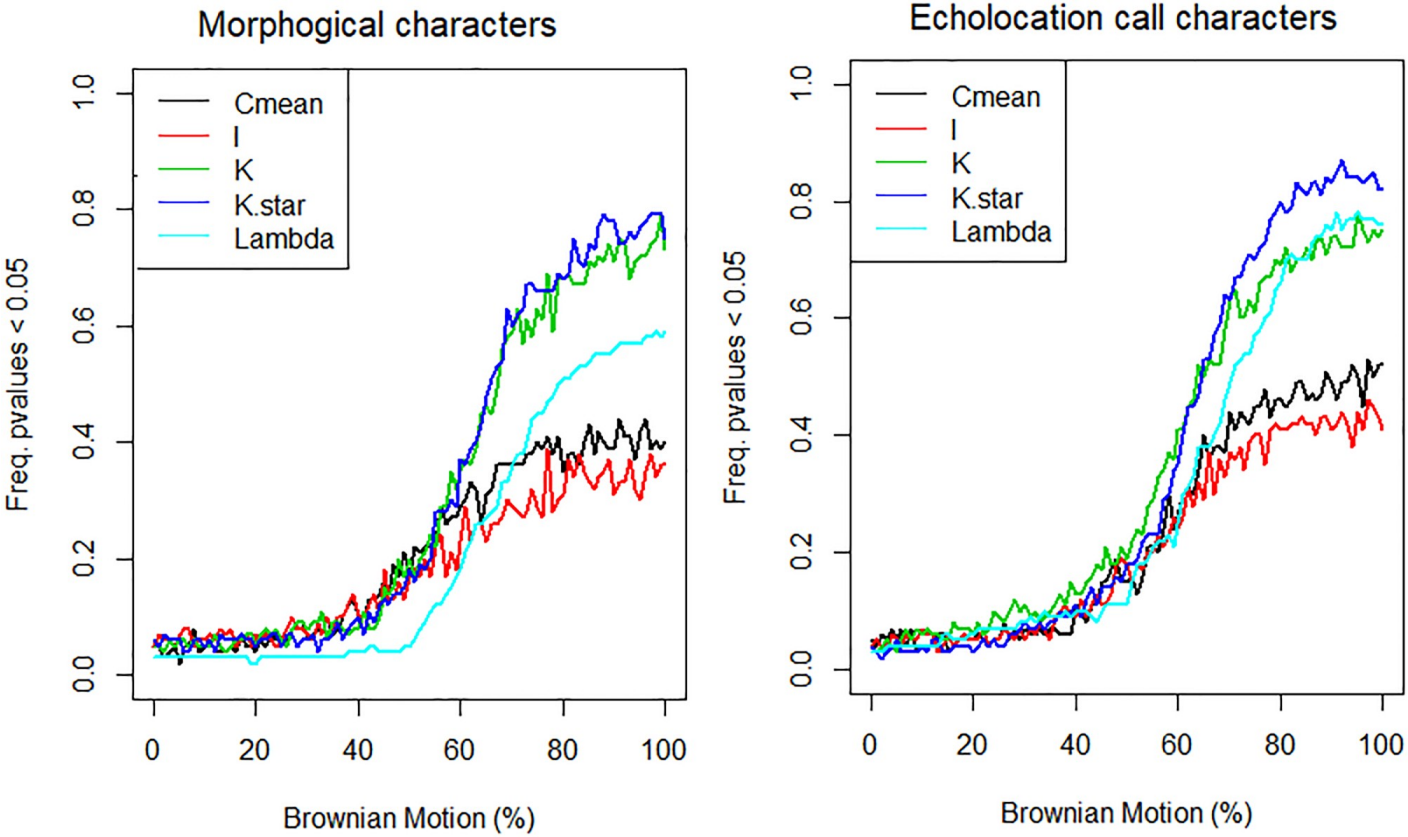
Plots of morphological and echolocation call characters showing the frequency of p values < 0.05 in five indices (Blomberg’s K and K*, Abouheif’s Cmean, Moran’s I, and Pagel’s Lambda) according to the proportion of Brownian Motion in the phylogeny of *Molossus*.

The indices used to calculate the stochastic Brownian Motion model obtained higher values for echolocation call traits, compared to morphological traits (Fig 6). The indices Blomberg’s K and K*, and Pagel’s Lambda have significant Brownian Motion for both data sets (p<0.05) indicating stochastically distribution of characters in 70%-80% of the phylogeny. The Abouheif’s Cmean and Moran’s I values showed significant mean values for the Brownian Motion model in 38% to 42% of the phylogeny for morphological characters and 40% to 55% for echolocation parameters.

**Fig 7:**
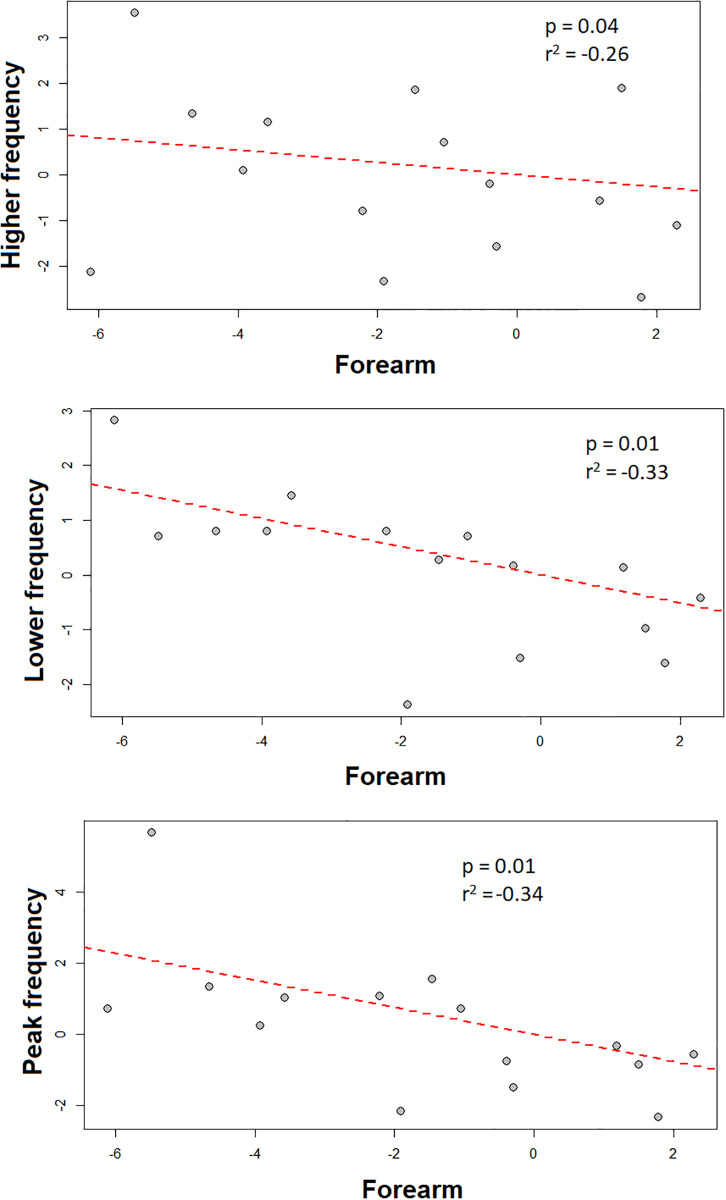
Independent contrasts between parameters of echolocation calls and morphological characters with significative p values (<0.05) for species of *Molossus*.

Significant negative relationships between independent contrasts of forearm size to the call parameters of minimum frequency (p = 0.01, r^2^ = -0.33), maximum frequency (p = 0.01, r^2^ = -0.34), and peak frequency (p = 0.04, r^2^ = -0.26) were found in regression analyses for all species (Fig 7). However, no significant relationship was found between any other morphological character and echolocation call parameters when the effect of phylogeny was removed (p>0.05). Among echolocation call variables, significant linear regression values were also found between call duration versus maximum frequency (p<0.001; R = 0.62), minimum frequency (p< = 0.02; R = 0.26), and peak frequency (p<0.01; R = 0.39).

Estimated ancestral reconstructions for each morphological and echolocation call character suggest that the ancestor of the *Molossus* lineage was probably of large body size (75%), had dichromatic dorsal hair with a wide pale band at the base (77%), long and thin upper incisors (94%), delicate and triangular occipital shape (70%), laterally directed infra orbital foramen (79%), and undeveloped sagittal crest in males (99%). The morphological ancestral reconstruction analysis suggests that the ancestor of *Molossus* was very similar to the extant species *M*. *sinaloae* and *M*. *alvarezi*, but that characteristics such as monochromatic fur, pincer-like incisors, and small body size are derived states that emerged more than once in the evolutionary history of the genus.

The echolocation call of the ancestral *Molossus* was likely short in call duration, less than 0.13 ms (82%), and the first harmonic had the highest energy (100%) (Table 3). The other ancestral states for echolocation call parameters could not be recovered with high probability. However, based on the relationship found between size and echolocation call frequencies, we hypothesize that the ancestral *Molossu*s also had a minimum frequency less than 26 kHz, a maximum frequency less than 35 kHz, and peak frequency less than 29 kHz. We could not predict the structure of the end slope in the ancestral node because this parameter does not seem to be correlated to size or to phylogeny.

**Table 3:** Likelihood probabilities of ancestral states of morphological and echolocation call characters in *Molossus*.

Node	1	2	3	4	5	6	7	8	9	10	11	12	13
**MORPHOLOGY**													
**1-Size**													
Very large	0.09	0.01	0	0	0	0	0	0	0	0	0	0	0
Large	0.75	0.76	0.55	0.48	0.12	0.46	0.55	0.57	0.54	0.67	0.59	0.54	0.51
Medium	0.11	0.18	0.34	0.51	0.88	0.43	0.45	0.41	0.46	0.33	0.41	0.46	0.49
Small	0.05	0.05	0.11	0.01	0	0.11	0.04	0.02	0	0	0	0	0
**2-Dorsal hair**													
Monochromatic	0.23	0.37	0.39	0.14	0.12	0.54	0.56	0.98	1	0.99	0.99	1	1
Dichromatic	0.77	0.63	0.61	0.86	0.88	0.46	0.44	0.02	0	0.01	0.01	1	1
**3-Upper incisors**													
Thin and enlogated	0.94	0.95	0.95	1	0.78	0.4	0	0	0	0	0	0	0
Pincerlike	0.03	0.04	0.06	0	0.22	0.6	1	1	1	1	1	1	1
Divergent tips	0.03	0.01	0	0	0	0	0	0	0	0	0	0	0
**4-Occiptal**													
Triangular	0.70	0.48	0.36	0.65	1	0.32	0.31	0.02	0.01	0.01	0.01	0	0
Rectangular	0.30	0.52	0.64	0.35	0	0.68	0.69	0.98	0.99	0.99	0.99	1	1
**5-Infraorbital foramen**													
Latterally	0.79	0.55	0.59	0.66	0.26	0.41	0.45	1	1	1	1	1	1
Forntally	0.21	0.45	0.41	0.33	0.74	0.59	0.55	0	0	0	0	0	0
**6-Sagittal crest**													
Absent	0	0	0	0	0	0	0	0	0	0	0	0	0
Undeveloped	0.99	0.99	0.95	1	1	0.67	0.66	0.01	0.01	0	0	0	0
Developed	0.01	0.01	0.05	0	0	0.33	0.34	0.99	0.99	1	1	1	1
**ECHOLOCATION CALLS**													
**1- Call duration**													
Long	0.07	0.04	0.01	0	0	0.01	0.01	0	0	0	0	0	0
Medium	0.11	0.12	0.11	0.01	0	0.28	0.62	0.99	1	1	1	1	0.99
Short	0.82	0.84	0.87	0.99	1	0.71	0.37	0.01	0	0	0	0	0.01
**2- Minimum Frequency**													
Low	0.54	0.55	0.53	0.44	0.11	0.51	0.49	0.5	0.69	0.59	0.57	0.58	0.56
High	0.46	0.45	0.47	0.76	0.89	0.49	0.51	0.5	0.31	0.41	0.43	0.42	0.44
**3-Maximum Frequency**													
Low	0.52	0.50	0.48	0.42	0.36	0.50	0.52	0.97	0.99	1	1	0.99	0.92
High	0.48	0.50	0.52	0.58	0.64	0.50	0.48	0.03	0.01	0	0	0.01	0.08
**4- Peak Frequency**													
Low	0.56	0.50	0.25	0.32	0.15	0.51	0.5	0.54	0.52	0.51	0.53	0.54	0.53
High	0.44	0.50	0.65	0.68	0.85	0.49	0.5	0.46	0.48	0.49	0.47	0.46	0.47
**5—High Energy Harmonic**													
First	1	1	1	1	1	1	1	1	1	1	1	0.99	0.99
Second	0	0	0	0	0	0	0	0	0	0	0	0.01	0.01
**6 -End Slope**													
Downward	0.56	0.52	0.49	0.41	0.79	0.52	0.43	0.41	0.88	0.65	0.72	0.76	0.75
Straight	0.44	0.48	0.51	0.59	0.21	0.48	0.57	0.59	0.11	0.35	0.28	0.24	0.25

Node numbers refer to the nodes reported in the phylogenetic tree in Fig 1.

## Discussion

We tested the hypotheses that, in *Molossus*, morphological and behavioural states are the result of evolutionary stasis and that morphology and echolocation calls evolved in concert. Distribution of character states most likely evolved by more than one modality. Morphology appears to evolve as a mosaic of adaptation, random drift, and stasis. However, call structure is independent of phylogeny in *Molossus*, evolving stochastically, and through local adaptations. Frequency of echolocation calls are negatively correlated with body size, and both characters seem to be evolving in concert, but variation in other morphological and behavioural characters among species are not correlated. Therefore, slight variation in both morphology and behaviour among species of the genus might evolve stochastically or via character displacement to avoid competition for resources in similar environments.

Our results show that morphology has a stronger evolutionary signal than behaviour, which is consistent with other studies. In a comparative study using a variety of organisms and traits [79], behaviour is less correlated with phylogeny than are morphology, life history, and physiological traits. Kamilar and Kooper [80] studied phylogenetic signals in primates and reported that although phylogenetic signal varies across traits and categories, behavioural characters had only a moderate to low correlation with the evolutionary branching pattern of the group. A correlation between some morphological and echolocation characters has also been reported in the literature [81, 82], which agrees with the findings found herein. A positive, but low, correlation between three individual morphological characters and phylogenetic distances suggests stability of those character states in the phylogeny, supporting the hypothesis that morphological stasis is occurring in some clades within *Molossus*. These characters are distributed in different morphological suites, including hair pattern, forearm length, and dentition, which suggest that stabilizing selection might be generalized across the phenotype within some groups of species. This pattern has also been observed in cryptic groups of ants [8], fishes [83], and lizards [17]. However, stasis localized in individual clades of the phylogeny might explain why similar species do not always form monophyletic groups. For example, body size is one of the most common traits used to characterize species of this genus, but it only has a correlation of 34% with phylogeny. Some closely related taxa may vary considerably in size from one another, and similar sized groups of bats may not be monophyletic, but instead consist of relatively distantly related species (Fig 3) [24, 84]. Thus, suites of characters traditionally used to define species and species groups in *Molossus* have led to a confused taxonomy.

Three other morphological characters that have been commonly used in species identification and systematic relationships in the genus (shape of the occipital bone, shape of the infraorbital foramen, and the relative development of the sagittal crest) are not strongly correlated with phylogeny. The apparent similarity among species in these character states seems to have arisen multiple times among phylogenetically divergent species, which explains the three non-monophyletic cryptic complexes within the genus (1—*M*. *molossus*—*M*. *milleri*—*M*. *verrilli*; 2—*M*. *currentium*—*M*. *bondae*; 3—*M*. *rufus*—*M*. *nigricans*—*M*. *fluminensis*). The lack of correlation between morphological and phylogenetic distances indicates that these traits are evolving stochastically or through convergence as adaptation to a particular environment or feeding guild [73, 85, 86], and may be more correlated with the use of different micro-ecological niches than with the phylogenetic history of a group [74–78, 87–91].

In contrast to vocal signals that are phylogenetically informative in birds and other mammals [90, 92–94], echolocation calls in *Molossus* did not appear to reflect phylogenetic patterns. Sensory convergence is considered to be one of the most important factors shaping the echolocation calls in bats [45, 46], and might be influenced by prey type and size [91], foraging strategy, and habitat selection [49, 95, 96]. Although species of *Molossus* have similar foraging strategies, they occupy an array of different habitats, such as tropical forests, savannahs, and urban areas [97], which might influence call structure. The prey perception hypothesis is unlikely to explain variability in frequencies since most bats have echolocation frequencies three times higher than required to detect their prey [49, 98] and larger bats can detect both small and large prey [99]. However, prey perception might act as a selective force in other echolocation parameters, such as call duration and shape of the terminal slope [100].

Species that rely on non-visual signals for orientation and foraging are more likely to be morphologically similar because the changes in these signals are not necessarily related to external morphology [101, 102]. However, in *Molossus*, correlations between body size and call frequencies suggest concerted evolution for these characters. Larger bats have lower call frequencies than smaller bats in agreement with the size-frequency hypothesis proposed by Jones [102]. According to Darwin [103] the length of the vocal cords is related to overall body size, and therefore larger animals usually emit lower fundamental frequencies, which could also explain the correlation. Studies have also suggested that cochlear size and shape is also related to body size in mammals [104], and can explain variation in echolocation in bats [105] and whales [106]. Jakobsen et al. [49] suggested that this relationship between body size and echolocation call frequencies might be explained instead by a constraint imposed by the need to achieve a high directionality of the call, which is not necessarily related to body size. According to these authors smaller bats have shorter jaws, which limit the maximum emitter size. Nevertheless, a recent study using 86 species of vespertilionid bats did not find support for the directionality hypothesis, and demonstrated that forearm size (a proxy for body size) is correlated with echolocation call peak frequency, which was consistent with our results [107].

No other echolocation call parameter measured in our study is correlated with morphological traits in *Molossus*. These results suggest that morphological and echolocation call characters, other than size and frequency, are evolving independently. However, the duration of the call appears to be correlated with frequency, whereby longer calls have lower frequencies. Species-specific adaptations are often connected with environmental factors, and the evolution of both morphological and behavioural traits can be influenced by micro-ecological selection pressures [108]. In bats, differences in call structure coupled with slight morphological variation might act to minimize competition [99], and thus not be correlated with phylogenetic histories of these species.

The low levels of phenotypic divergence found within the three polyphyletic cryptic species complexes show that unequal trait diversification has evolved mostly through local adaptation or random walk. Indeed, the Brownian Motion model suggests that a significant fraction of both character sets is evolving stochastically, but not all the evolution of these character can be explained by random walks. These results suggest that evolutionary processes other than stasis and Brownian Motion, such as recent adaptation, might affect the evolution of those traits. These patterns explain why so many species within the genus are morphologically and behaviorally similar, regardless of their level of phylogenetic divergence.

## Supporting information

S1 Appendix Morphological data. (XLS)

S2 Appendix Echolocation data. (XLS)
